# Inhibition of heat shock protein 90 in the mouse spinal cord enhances opioid antinociception via the upregulation of dual specificity protein phosphatase 15 (DUSP15) in CGRP neurons

**DOI:** 10.3389/fphar.2026.1734504

**Published:** 2026-10-19

**Authors:** Katherin A. Gabriel, Jessica L. Bowden, John M. Streicher

**Affiliations:** 1 Department of Pharmacology, College of Medicine, University of Arizona, Tucson, AZ, United States; 2 Comprehensive Center for Pain and Addiction, University of Arizona, Tucson, AZ, United States

**Keywords:** antinociception, calcitonin gene-related peptide (CGRP), dual specificity protein phosphatase 15 (DUSP15), heat shock protein 90 (hsp90), opioid

## Abstract

In an extended series of studies, we have shown that Heat shock protein 90 (Hsp90) has a novel role in regulating opioid signal transduction and antinociception in both the brain and spinal cord. Inhibition in the spinal cord enhances opioid antinociception, which led to the finding that an isoform-selective Hsp90 inhibitor could improve opioid therapy, both enhancing pain relief and reducing side effects. However, the signaling mechanisms for this activity are still not fully understood. We thus identified Dual Specificity Phosphatase 15 (DUSP15) from an earlier proteomic study as a protein enhanced in the spinal cord after Hsp90 inhibition. Using male and female CD-1 mice, we confirmed that DUSP15 is upregulated in the dorsal horn of the spinal cord after Hsp90 inhibitor (17-AAG) treatment. Using CRISPR and siRNA-mediated knockdown, we showed that DUSP15 was necessary for the enhanced morphine antinociception after Hsp90 inhibitor treatment in both the tail flick and post-surgical paw incision pain models. Using immunohistochemistry (IHC) and behavioral tools, we placed DUSP15 upstream of both ERK MAPK and AMPK in the opioid signaling cascade. We then used IHC to demonstrate that DUSP15 upregulation occurs at least in part in CGRP neurons, and using cell-selective CRISPR, showed that DUSP15 in CGRP neurons is necessary for the enhanced antinociception observed with spinal Hsp90 inhibitor treatment. Together these studies further expand the known mechanisms by which Hsp90 regulates opioid signal transduction and pain relief and provide basic science insight into a potential strategy to improve opioid therapy using Hsp90 inhibitors.

## Introduction

Mu opioid receptor (MOR) signal transduction is a highly complex process, incorporating elements of subcellular localization (e.g., chaperones), microdomain organization (e.g., scaffold proteins), and contingent activity regulated by different cell types spatially distributed among the nodes of a neuronal circuit (Waheed and Jones, 2002; Al-Hasani and Bruchas, 2011; Streicher, 2019). Previous work has shown that MOR signal transduction can be manipulated or exploited in order to improve opioid therapeutic outcomes, such as with biased agonists (Viscusi et al., 2016; Schmid et al., 2017). Despite these advances, however, much of the full MOR signal transduction cascade is unknown, especially in terms of linking signaling outcomes to antinociception and side effects.

To this end, we’ve engaged in a long-term investigation of the signaling regulator and chaperone Heat shock protein 90 (Hsp90). Hsp90 has numerous client proteins and physiological roles, from localizing signaling proteins to specific domains within the cell to regulating the proper activation of signaling kinases (Ota et al., 2010; Li and Buchner, 2013; Streicher, 2019). We linked Hsp90 to opioid signaling and pain relief with our finding that Hsp90 *promotes* MOR signaling and antinociception in the brain via ERK MAPK activity (Lei et al., 2017; Stine et al., 2020). In contrast, we found that Hsp90 *restrains* MOR signaling in the spinal cord, so that spinal Hsp90 inhibition leads to enhanced opioid antinociception (Duron et al., 2020). We further found that this enhanced antinociception can form the basis of an opioid dose-reduction strategy to use Hsp90 isoform-selective (Hsp90β, Grp94) inhibitors to improve the opioid therapeutic index, enhancing pain relief while reducing side effects (Lei et al., 2019; Duron et al., 2024).

We’ve also made progress in identifying the signaling cascade underlying this enhancement in opioid antinociception. We found that dendritic ERK and RSK1/2 in the spinal cord dorsal horn were two necessary kinase proteins that promote antinociception (Duron et al., 2020). Adding further complexity to this cascade, we identified AMPK as a negative feedback regulator of MOR signaling upstream of ERK that is disabled by Hsp90 inhibition. This blockade of AMPK signaling specifically in Calcitonin Gene-Related Peptide (CGRP) neurons in the dorsal horn was sufficient for enhanced opioid antinociception (Gabriel and Streicher, 2023). Through these studies, we have begun to identify specific signaling proteins in the Hsp90-MOR cascade, identify their order of activation, and locate them in a spatially distributed and integrated molecular-cell circuit in the spinal cord. However, these studies represent only the first few steps, and much more of this impactful cascade remains to be uncovered.

To do so, we examined in this study Dual Specificity Phosphatase 15 (DUSP15), which we found was upregulated in our earlier proteomic study of mouse spinal cord after Hsp90 inhibitor treatment (Duron et al., 2020). Not much is known about DUSP15; it’s been shown to regulate Notch (Bhore et al., 2021) and Jak1/STAT3 (Kikkawa et al., 2024) signaling, and perhaps most relevant, it’s been found to negatively regulate morphine-associated contextual memory in the Nucleus Accumbens (Qiao et al., 2021). However, one paper did report that DUSP15, a phosphatase, promotes ERK activation in Schwann cells (Rodriguez-Molina et al., 2017). Our observed upregulation of DUSP15 (Duron et al., 2020) and our finding that ERK promotes enhanced antinociception after Hsp90 inhibition (Duron et al., 2020) suggested a hypothesis whereby DUSP15 activates ERK and thus enhanced opioid signaling after spinal Hsp90 inhibition. Our observations support this hypothesis, and suggest a DUSP15-AMPK-ERK signaling cascade that promotes opioid antinociception after Hsp90 inhibition in the spinal cord.

## Materials and methods

### Drugs

17-AAG (#AAJ66960MC), a non-isoform-selective Hsp90 inhibitor, DAMGO (#11711), and Dorsomorphin dihydrochloride (#30–935–0) were purchased from Fisher Scientific (Waltham, MA). Morphine sulfate pentahydrate was obtained through the National Institute on Drug Abuse Drug Supply Program and distributed through the Research Triangle Institute. 17-AAG was prepared as a stock solution in DMSO, while DAMGO and Dorsomorphin were prepared as stock solutions in water. Morphine was prepared fresh for each experiment in USP saline. Powders were stored as recommended by the manufacturer, and stock solutions stored at −20 °C. Appropriate vehicle controls were used for each experiment: 1% DMSO in water for 17-AAG intrathecal (i.t.) injections; water for DAMGO and Dorsomorphin i. t. Injections; USP saline for systemic morphine injections. The doses used for all drugs were established in our previous work (Lei et al., 2017; Duron et al., 2020; Gabriel and Streicher, 2023).

### CRISPR DNA constructs and siRNA

A CRISPR gene editing construct was obtained from Genecopoeia (Rockville, MD) as all-in-one CRISPR clone targeting both variants for mouse DUSP15 (#CS-MCP273659-CG12-3B). We also used a second construct in the pCRISPR-CG12 vector backbone with the mouse CGRP (*Calca*) promoter (#MPRM31284) used to selectively drive expression of the Cas9 gene in CGRP-expressing cells (#CS-MCP001980-CG12-1–10). We also used a universal negative control vector that expresses all the same elements with a non-targeting gRNA (#CCPCTR01-CG01-B). Each DNA vector was amplified for use using standard bacterial transformation, with an endotoxin-free maxi-prep kit to reduce inflammation upon injection (#12362, Qiagen, Germantown MD). DUSP15 siRNA was obtained from Integrated DNA Technologies (Coralville, IA) to target mouse DUSP15 mRNA along with a universal negative control sequence siRNA (#51-01-14-04). The DUSP15 siRNA sequence was: 5′-AAU GGC AUG ACC AAG GUA CUU CCT G-3’.

### Animals

Male and female CD-1 mice in age-matched controlled cohorts from 5 to 8 weeks of age were used for all experiments and were obtained from Charles River Laboratories (Wilmington, MA). Male and female mice were used in approximately equal numbers in each experiment; no sex differences were observed, so the male and female cohorts were combined for the data shown. CD-1 (a.k.a. ICR) mice are commonly used in opioid research and in our previous work (Duron et al., 2020; Gabriel and Streicher, 2023) as a line with a strong response to opioid drugs. Mice were recovered for a minimum of 5 days after shipment before being used in experiments. The mice were kept in an AAALAC accredited vivarium at the University of Arizona under temperature control and 12-h light/dark cycles with food (standard lab chow) and water available *ad libitum*. No more than five mice were kept in a cage. The animals were monitored daily, including after surgical procedures, by trained veterinary staff. All experiments performed were in accordance with IACUC-approved protocols at the University of Arizona and according to the guidelines of the NIH Guide for the Care and Use of Laboratory Animals handbook.

### Behavioral experiments

Animal cohorts were randomly block assigned to treatment group by cage. Prior to any behavioral experiment or testing, the animals were brought to the testing room in their home cages for at least 1 h for acclimation. Testing always occurred within the same approximate time of day between experiments during the light (inactive) cycle, and environmental factors (noise, personnel, and scents) were minimized. All testing apparatus (mesh boxes, etc.) were cleaned between uses. The experimenter was blinded to treatment group by another laboratory member delivering coded drug vials, which were then decoded after collection of all data.

### Tail flick assay

Pre-injection tail-flick baselines were determined using a 52 °C warm water tail flick assay with a 10 s cutoff time (Lei et al., 2017; Gabriel and Streicher, 2023). All i. t. Injections were performed as we previously reported (Duron et al., 2020; Gabriel and Streicher, 2023; Duron et al., 2024). For CRISPR experiments, 4.5 µg of DNA was complexed with GenJet *in vivo* transfection reagent (#50-485-2 from Fisher Scientific) according to the manufacturer’s instructions and injected i. t. twice a day from days 1–3. On day 9, baseline measurements were taken again, then the mice were injected with 0.5 nmol 17-AAG or vehicle control, i. t., with a 24 h recovery. On day 10, baseline measurements were taken a final time, then the mice were injected with 3.2 mg/kg morphine by the subcutaneous (s.c.) route with a 2-h tail flick time course measured. This approach is the same as we previously reported (Gabriel and Streicher, 2023; Duron et al., 2024).

For the siRNA experiments, baseline measurements were taken, then mice were injected i. t. with 12.1 μM concentration (5 µL volume) of siRNA complexed with *in vivo* transfection reagent as above once a day for two days. On day 3, mice were baselined again then injected with 0.5 nmol of 17-AAG or vehicle control (i.t.). On day 4, the final baseline was taken, then 3.2 mg/kg morphine was injected s. c. and tail flick latencies were determined over a 2-h time course.

### Paw incision and mechanical allodynia

Mechanical thresholds were determined prior to surgery using calibrated von Frey filaments (Ugo Basile, Varese, Italy) with the up-down method and four measurements after the first response per mouse (Lei et al., 2017; Duron et al., 2020; Stine et al., 2020; Gabriel and Streicher, 2023; Duron et al., 2024). Measurements were taken in a homemade apparatus with Plexiglas walls and ceiling and a wire mesh floor (3″ W x 4″ L x 3″ H with 0.25″ wire mesh). The surgery was then performed by anesthesia with ∼2% isoflurane in standard air, preparation of the left plantar hind paw with iodine and 70% ethanol, and a 5-mm incision made through the skin and fascia with a no. 11 scalpel. The muscle was elevated with curved forceps leaving the origin and insertion intact, and the muscle was split lengthwise using the scalpel. The wound was then closed with 5–0 polyglycolic acid sutures (Lei et al., 2017; Gabriel and Streicher, 2023).

The CRISPR injections were performed as above for the tail flick experiments. The mice then recovered, with the paw incision surgery performed on day 9 along with i. t. Injection of 17-AAG or vehicle control as above. On day 10, 3.2 mg/kg morphine was injected s. c. and von Frey measurements taken over a 3-h time course. Baseline measurements were taken before CRISPR treatment on day 0, before surgery and 17-AAG injection on day 9, and before morphine injection on day 10.

The siRNA injections were also performed as above for the tail flick assay. On day 3, mice were injected with 0.5 nmol of 17-AAG or vehicle control i. t., and also received the paw incision surgery. On day 4, morphine (3.2 mg/kg, s. c.) was injected and mechanical threshold measurements were determined over a 3-h time course. Baseline measurements were similarly taken on day 0, before surgery on day 3, and before morphine on day 4.

### Antibodies

The antibodies used were: pERK (Cell Signaling #4370S, lot 12, rabbit, 1:1,000 for IHC), DUSP15 (Sigma-Aldrich #SAB1401989-10, Lot 09,112, 1:50 for IHC, 1:1,000 for Western), CGRP (Abcam #ab81887, 1:200 for IHC), pAMPK (Invitrogen #PA5-37821, lot XC3525642, 1:200 for IHC), GαM680 Secondary (LI-COR #926–68020, lot C50721-02, 1:10,000 for Western), GαR800 Secondary (LI-COR #926–32211, lot C50602-05, 1:10,000 for Western), GαM-Alexa594 Secondary (Invitrogen #A11032, lot 1,985,396, 1:500 for IHC), and GαR-Alexa488 Secondary (Invitrogen #A11034, lot 2,110,499, 1:200 for IHC).

### Immunohistochemistry

Our IHC protocol was substantially similar to our previous work (Duron et al., 2020; Gabriel and Streicher, 2023). Perfusions were performed on treated mice with cold PBS, followed with cold 4% paraformaldehyde in PBS. Shortly after the perfusions were complete, fixed spinal cords were extracted and immediately placed in cold 4% paraformaldehyde for ∼6 h. Spinal cords were then placed in 15% sucrose in PBS overnight, followed by 30% sucrose in PBS overnight. Spinal cords were then flash frozen in OCT compound using liquid nitrogen and sectioned with a Microm HM525 cryostat at a thickness of 20 µm between the L5 and L6 vertebrae region and mounted on Surgipath X-tra microscope slides. Spinal cord sections were rehydrated in PBS in preparation for free float staining. Samples were incubated in an endogenous peroxidase blocking buffer consisting of 60% methanol and 0.3% hydrogen peroxide in PBS at room temperature for 30 min and then washed with PBST (PBS +0.1% Tween20). They were then incubated in 5% goat serum, 1% bovine serum albumin in PBST at room temp for 1 h. Samples were then incubated with primary antibodies in 1.5% goat serum and 1% bovine serum albumin in PBST at 4 °C overnight. Samples were then washed with PBST and then incubated with secondary antibodies in 5% goat serum and 1% bovine serum albumin in PBST at room temp for 1 h in the dark. Samples were prepared as instructed by the manufacturer using the VECTASTAIN Elite ABC horseradish peroxidase kit (#PK-6101) and TSA Plus Fluorescein evaluation kit (#NEL741E001KT), both from PerkinElmer. Stained spinal cord sections were then mounted onto slides with Novus FluorEver. Sections were imaged using an Olympus BX51 microscope equipped with a Hamamatsu C8484 digital camera. Images were analyzed using ImageJ. Dorsal horn regions were selected, and average mean intensities were measured and normalized to vehicle controls.

### Western blotting and analysis

Mouse spinal cord protein lysates were prepared using our previously published protocol (Lei et al., 2017) and quantified with a BCA protein quantitation assay using the manufacturer’s protocol (Bio-Rad). The protein was run on precast 10% Bis-Tris Bolt gels (Fisher Scientific #NW00100BOX) using the Bolt gel apparatus and following the manufacturer’s instructions. The gels were transferred to nitrocellulose membrane (Bio-Rad) using a wet transfer system (30 V, minimum of 1.5 h on ice). The blots were blocked with 5% nonfat dry milk in Tris-Buffered Saline (TBS) and incubated with primary antibody in 5% bovine serum albumin in TBS +0.1% Tween20 (TBST) overnight rocking at 4 °C. The blots were then washed three times for 5 min in TBST, incubated with secondary antibody (see above) in 5% milk in TBST for 1 h of rocking at room temperature, washed again, and imaged with an Azure Sapphire infrared imaging system. Some blots were then stripped with 25 mM glycine-HCl and 1% SDS, pH 2.0, for 30–60 min of rocking at room temperature prior to being washed and re-exposed to primary antibody. The resulting image bands were quantified using Scion Image (based on NIH Image). All images were quantified in the linear signal range, which is easier to ensure because the Azure imager is a dynamic imager that allows for fine control of exposure. DUSP15 signal was normalized to GAPDH. The normalized intensities were further normalized to a negative control present on the same blot.

### RNA extraction and reverse transcription

RNA was extracted from frozen spinal cords using TRIzol reagent (Invitrogen #15596026) following the supplier instructions. At the end of the extraction procedure, RNA pellets were diluted in 40 µL of nuclease-free water. The concentration of RNA samples was measured using a NanoDropND-1000 spectrometer (NanoDrop Technologies) and overall quality was assessed by measurement of the 260/280 nm ratio. A total amount of 1 µg of RNA was used as the template for reverse transcription using the Applied Biosystems High-Capacity RNA-to-cDNA Kit (Invitrogen #4387406). The mixture was incubated following the procedure: i) 60 min at 37 °C, ii) 5 min at 95 °C, iii) 4 °C hold.

### Quantitative PCR

Real-time PCR was performed in a 96-well plate format, each well containing 0.6 µL of each of the primers, 1 µL of cDNA diluted 1:10, and 12.5 µL of Power SYBR Green PCR Master Mix (#4368702 from Applied Biosystems). Each reaction was run in triplicate on a Bio-Rad iCycler qPCR system with the following protocol: i) 10 min at 95 °C, ii) 15 s at 95 °C iii) 1 min at 60 °C (45 cycles of ii-iii), iv) 10 min at 72 °C, followed by a melt curve analysis. Target mRNA abundance was calculated and normalized to GAPDH quantity, and further normalized to the control group on each plate. The melt curve analysis was used to confirm one product per reaction. Our RNA extraction and qPCR protocol was also reported in our previous work (Lei et al., 2020). The primer sequences used were: mDUSP15-F: 5′-TTT AGG CAG CAG CTC GAG GA-3’; mDUSP15-R: 5′-AGA AGA AAG TCT GCT TGA CGC-3’; GAPDH-F: 5′-TCC TGC ACC ACC AAC TGC TTA G-3’; GAPDH-R: 5′-GAT GAC CTT GCC CAC AGC CTT G-3’.

### Data analysis

All data were reported as the mean ± SEM and normalized where appropriate as described above to various control groups. The behavioral data were reported raw without maximum possible effect (MPE) or other normalization. Biological and technical replicates are described in the Figure Legends. Comparisons between two groups (proteomic analysis, Western blot, IHC) were performed by unpaired 2-tailed *t* tests. One variable comparisons between more than two groups (mRNA quantity, pAMPK IHC) were performed by 1-Way ANOVA with Tukey’s *post hoc* test. Behavioral time course data were compared by Repeated Measures (RM) 2-Way ANOVA with Dunnett’s *post hoc* test. In all cases, significance was defined as *p* < 0.05. All graphing and statistical analyses were performed using GraphPad Prism 8.3 (San Diego, CA).

## Results

Male and female mice were used in every experiment. No significant sex differences were detected, so the sexes were combined in each group shown below.

### Spinal Hsp90 inhibition boosts DUSP15 expression to promote opioid antinociception

We analyzed the results of our previously published proteomic study on mouse spinal cord tissue treated with 17-AAG, an Hsp90 inhibitor that is non-selective between Hsp90 isoforms, in order to find potential new Hsp90/MOR signaling regulators (Duron et al., 2020). We found that the phosphatase DUSP15 was upregulated by 17-AAG treatment (Figure 1A). While little is known of DUSP15 signaling, it had been previously shown to promote ERK MAPK activity (Rodriguez-Molina et al., 2017), which we found is part of the Hsp90/MOR cascade (Duron et al., 2020), and in addition was linked to morphine-associated contextual memory in the nucleus accumbens (Qiao et al., 2021). We thus hypothesized that DUSP15 upregulation contributes to enhanced opioid antinociception after spinal Hsp90 inhibition.

**FIGURE 1:**
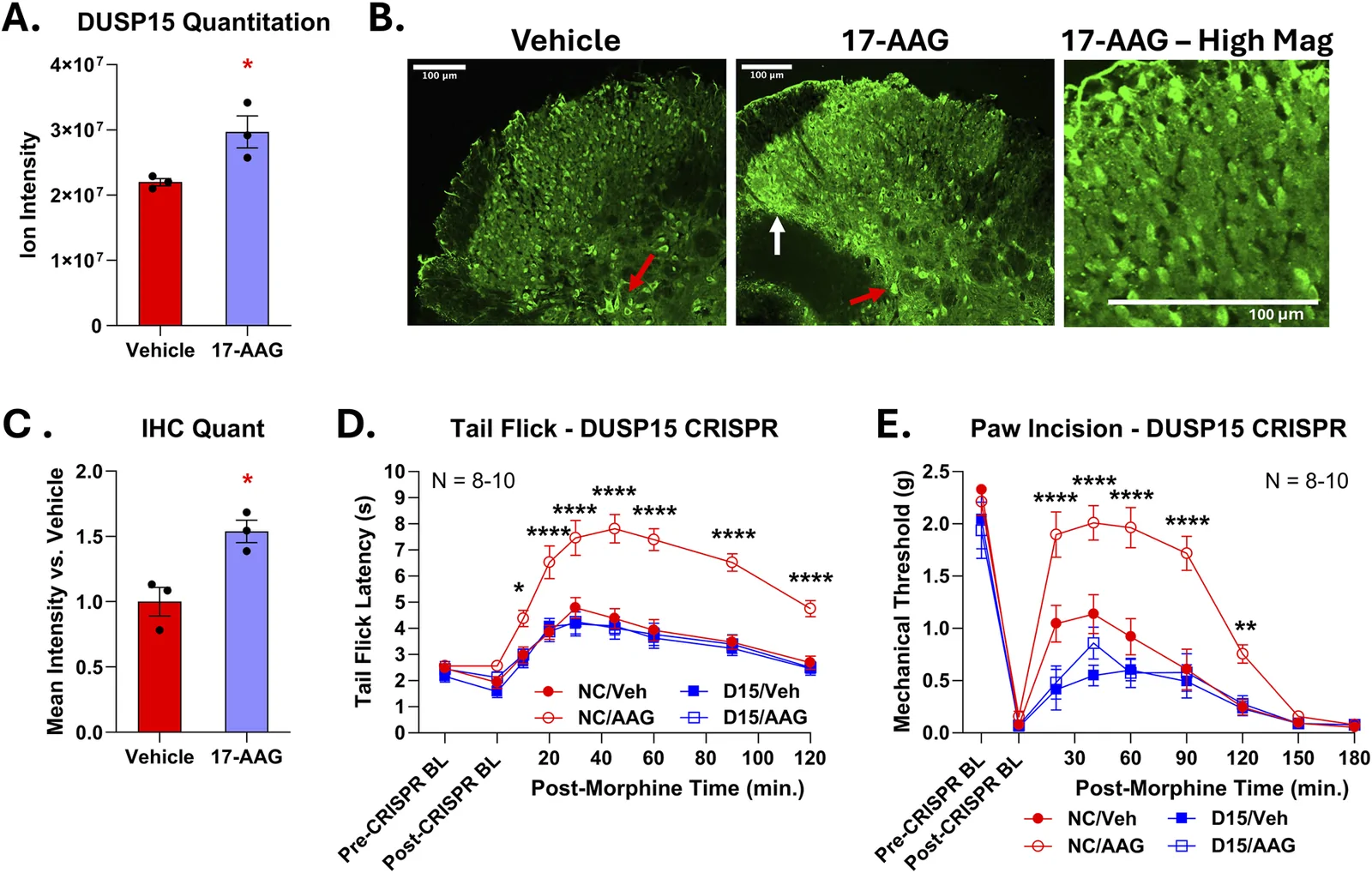
Spinal cord Hsp90 inhibition with 17-AAG upregulates DUSP15, which is necessary for enhanced morphine antinociception. Data represented as the mean ± SEM. **(A)** Female CD-1 mice, vehicle or 0.5 nmol 17-AAG injected i.t., 24 h, then spinal cords analyzed by proteomics. N = 3 mice/group. DUSP15 protein levels shown, which are upregulated by 17-AAG treatment. * = *p* < 0.05 by unpaired 2-tailed *t*-test. Data taken from the experiment reported in (Duron et al., 2020). **(B)** Male and female CD-1 mice, vehicle or 0.5 nmol 17-AAG injected i.t., 24 h, then spinal cords analyzed by IHC. N = 3 mice/group (1 male, 2 females each) with 3 slices per mouse, representative images shown. Dorsal horn regions shown. DUSP15 signal (green) is visible as a cell body/cytoplasmic stain that is present in the deep layers of the dorsal horn in both treatment groups (red arrows). After Hsp90 inhibition with 17-AAG, DUSP15 signal is visibly upregulated in the superficial dorsal horn (white arrow). High magnification image highlights the cell body stain in the superficial layers. **(C)** Quantification of the IHC experiment in B. * = *p* < 0.05 by unpaired 2-tailed *t*-test. Confirms DUSP15 upregulation after 17-AAG treatment. **(D)** Male and female CD-1 mice treated with Negative Control (NC) or DUSP15-targeted CRISPR (see Methods). On day 9, mice injected with vehicle or 0.5 nmol 17-AAG, i.t., 24 h, followed by 3.2 mg/kg morphine s. c. and a tail flick time course performed. Pre- and post-CRISPR baseline (BL) measurements also taken. N = 8–10 mice/group, performed in 2 technical replicates. *, **** = *p* < 0.05, 0.0001 vs. same time point NC/Veh group by RM 2-Way ANOVA with Dunnett’s *post hoc* test. **(E)** Male and female CD-1 mice treated with CRISPR and veh/17-AAG as in **(D)**, except the paw incision surgery was performed on all groups concurrent with veh/17-AAG injection on day 9. On day 10, 3.2 mg/kg morphine was injected s. c., and a time course performed, measuring the mechanical threshold using von Frey filaments. BL measurements also taken. N = 8–10 mice/group, performed in 2 technical replicates. **, **** = *p* < 0.01, 0.0001 vs. same time point NC/Veh group by RM 2-Way ANOVA with Dunnett’s *post hoc* test. In both cases, DUSP15 knockdown reversed the enhanced morphine antinociception caused by Hsp90 inhibition, without altering the baseline (D15/Veh) response.

To test this hypothesis, we first confirmed that DUSP15 was upregulated in the spinal cord after Hsp90 inhibitor (17-AAG) treatment. Using IHC, we found clear DUSP15 expression in the apparent cytoplasmic compartment that was especially prominent in the deep region of the dorsal horn of the spinal cord, in the transition zone close to the intermediate horn (Figure 1B, red arrows). After spinal Hsp90 inhibition, we observed a visual increase in the superficial layers of the dorsal horn similarly localized to cell bodies in a cytoplasmic pattern (Figure 1B, white arrow). The superficial layers of the dorsal horn are strongly implicated in pain and opioid regulation (Heinricher et al., 2009; Wang et al., 2018), providing a plausible link between DUSP15 upregulation and opioid signaling modulation. This visual observation was confirmed by quantitation (Figure 1C), which showed a near-identical increase in magnitude to the proteomic analysis in Figure 1A.

To definitively link DUSP15 to opioid receptor activity, we knocked down DUSP15 expression in the spinal cord using CRISPR *in vivo* transfection in adult wild type mice (see Methods, (Lei et al., 2019; Gabriel and Streicher, 2023; Duron et al., 2024); no inhibitors or other ligands available for DUSP15). This was combined with spinal 17-AAG treatment (0.5 nmol, i. t., established in (Lei et al., 2017; Duron et al., 2020; Gabriel and Streicher, 2023)) and morphine (3.2 mg/kg, s. c.) in the tail flick (Figure 1D) and post-surgical paw incision (Figure 1E) pain models. In both cases, spinal Hsp90 inhibition with 17-AAG approximately doubled the antinociceptive efficacy of morphine, as expected (Duron et al., 2020; Gabriel and Streicher, 2023; Duron et al., 2024); also in both cases, DUSP15 knockdown fully reversed the antinociceptive enhancement back down to baseline (NC/Veh group) levels, but no further (Figures 1D,E). DUSP15 knockdown alone had no impact on morphine antinociception without 17-AAG treatment (NC/AAG group). The effectiveness of the CRISPR treatment was validated by Western blot (Supplementary Figures S1,S2).

Since CRISPR gene editing can have off-target effects, we confirmed our findings with a second approach by *in vivo* spinal transfection of an siRNA targeted at DUSP15 mRNA (see Methods). The experiment was designed analogously to the CRISPR experiments above, and in the tail flick pain model, DUSP15 siRNA similarly reversed the enhanced morphine antinociception caused by 17-AAG treatment while having no effect on its own (Figure 2A). The effectiveness of the siRNA treatment was validated by qPCR (Figure 2B). These two complementary approaches validate our observations and suggest that DUSP15 is upregulated to promote enhanced opioid antinociception after spinal Hsp90 inhibition.

**FIGURE 2:**
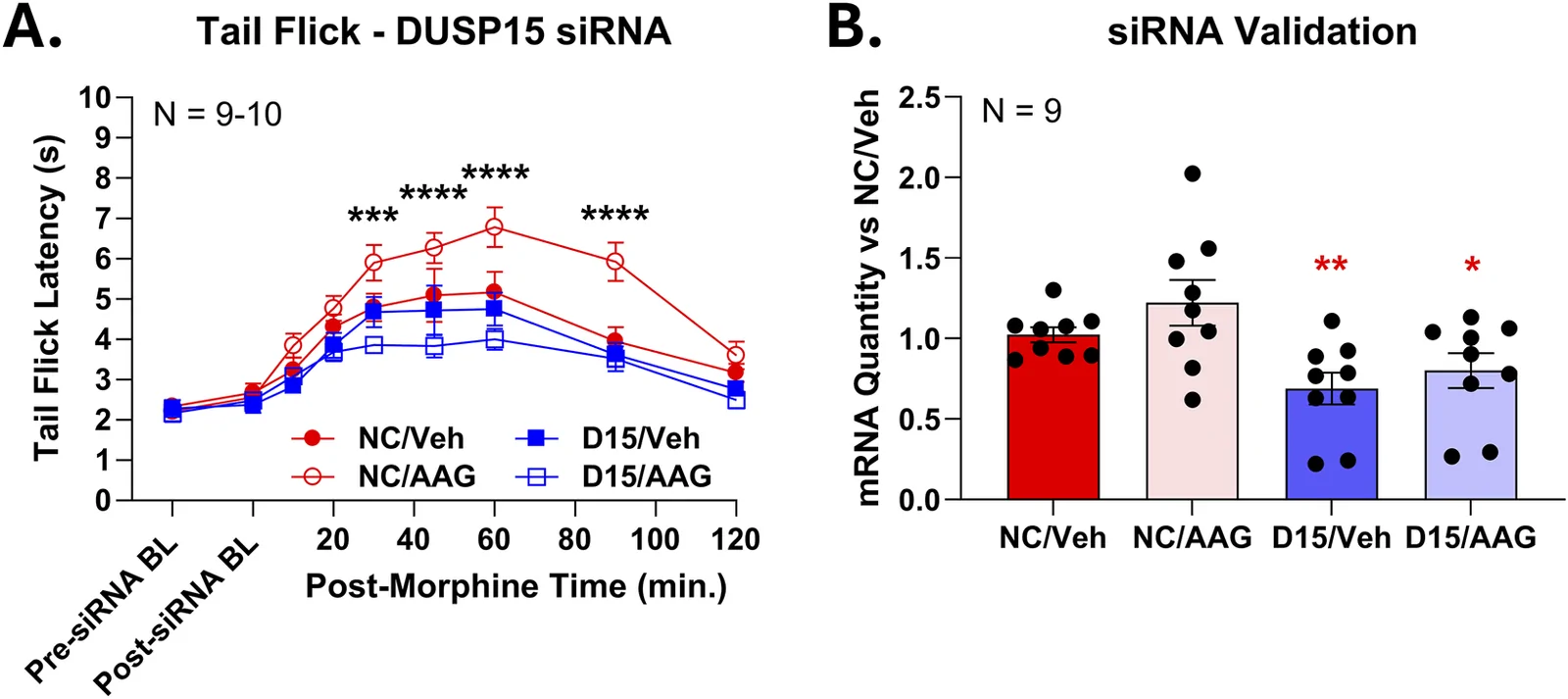
Confirmation of the role of DUSP15 using siRNA. Data represented as the mean ± SEM. **(A)** Male and female CD-1 mice were transfected with Negative Control (NC) or DUSP15-targeted siRNA by the i.t. route (see Methods). On day 3, the mice were injected with vehicle or 0.5 nmol 17-AAG i.t., 24 h, followed by 3.2 mg/kg morphine s. c. and a tail flick time course was performed. Baseline (BL) measurements were also taken. N = 9–10 mice/group performed in 2 technical replicates. ***, **** = *p* < 0.001, 0.0001 vs. same time point NC/Veh group by RM 2-Way ANOVA with Dunnett’s *post hoc* test. **(B)** The spinal cords from the treated mice in **(A)** were analyzed for *Dusp15* mRNA levels by RT-qPCR. The resultant values were normalized to a GAPDH control from the same sample on the same plate, and further normalized to the NC/Veh group. *, ** = *p* < 0.05, 0.01 vs. NC/Veh group by 1-Way ANOVA with Tukey’s *post hoc* test. Overall, the experiment confirmed and validated the DUSP15 CRISPR approach above.

### Placement of DUSP15 in the Hsp90/MOR signal transduction cascade

Our experiments showed that DUSP15 was upregulated and contributed to enhanced opioid pain relief, but did not demonstrate key mechanistic details. We thus first sought to place DUSP15 within the Hsp90/MOR signal transduction cascade, which to date includes ERK MAPK (Duron et al., 2020) and AMPK (Gabriel and Streicher, 2023). To do so, we combined spinal Hsp90 inhibition and opioid stimulation (17-AAG/DAMGO) with the DUSP15 CRISPR approach from above and measured phosphorylated ERK MAPK (pERK) in the spinal cord using IHC. The negative control (NC) CRISPR treatment showed visually intense pERK staining in a reticular pattern in the superficial layers of the dorsal horn (Figure 3A); this was similar to our previous results (Duron et al., 2020). DUSP15 CRISPR treatment abolished this effect (Figure 3A), which was confirmed by quantitation (Figure 3B). This observation suggests that DUSP15 is upstream of ERK in the Hsp90/MOR cascade, which would fit the previous DUSP15-ERK signaling literature (Rodriguez-Molina et al., 2017). In addition, we noted that the reticular pattern of ERK (colocalized with the dendritic marker MAP2 (Duron et al., 2020)) was different from the cell body pattern of DUSP15 in Figure 1. This suggests the molecular signaling circuit is spread across space and may occur across multiple cells.

**FIGURE 3:**
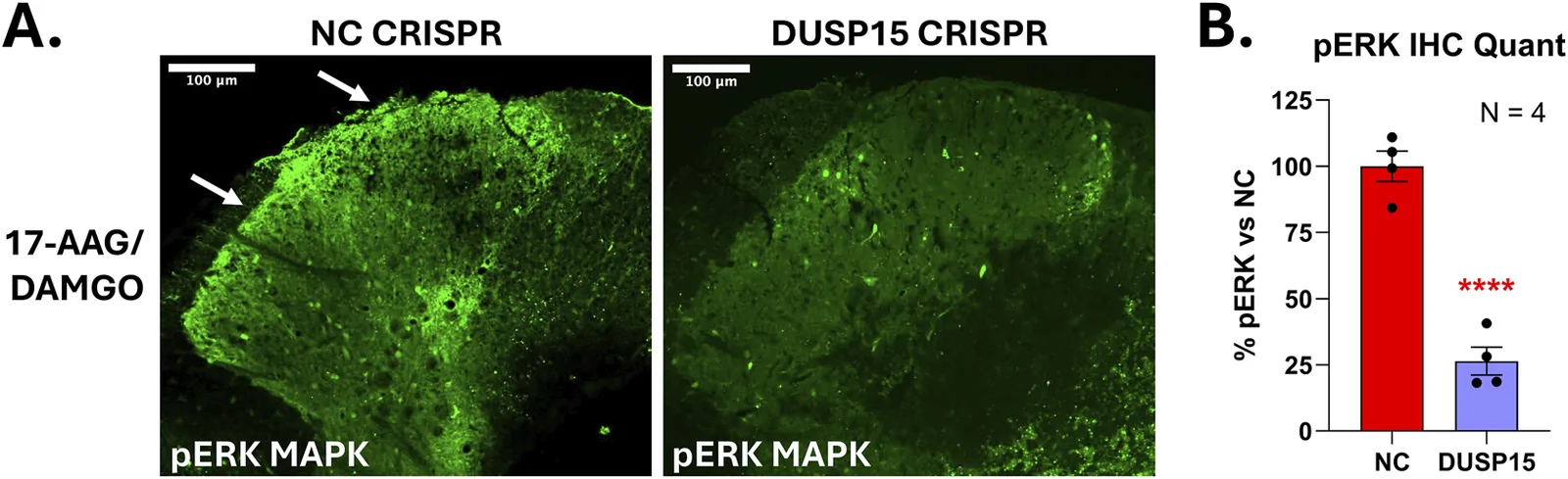
Placement of DUSP15 upstream of ERK MAPK in the Hsp90-MOR signaling cascade. **(A)** Male and female CD-1 mice treated with Negative Control (NC) or DUSP15 CRISPR by the i.t. route (see Methods). On day 9, the mice were injected with 0.5 nmol 17-AAG i. t., 24 h, then 0.1 nmol DAMGO i.t., 10 min, then sacrificed and their spinal cords analyzed by IHC. N = 4 mice/group with 2 sections per mouse analyzed, representative images shown. The dorsal horn region is also shown. Phosphorylated ERK (pERK) signal (green) is visible in the NC CRISPR group as a reticular pattern in the superficial layers of the dorsal horn, as expected (white arrows (Duron et al., 2020; Gabriel and Streicher, 2023). When DUSP15 was knocked down, this visual upregulation of ERK activity was lost, suggesting that DUSP15 is upstream of ERK in the Hsp90-MOR cascade. **(B)** The visual observations from **(A)** were confirmed by quantitation. Data represented as the mean ± SEM, and were further normalized to the NC group. **** = *p* < 0.0001 by unpaired 2-tailed *t*-test.

We similarly tested the relationship between DUSP15 and phosphorylated AMPK (pAMPK). As described in our prior work (Gabriel and Streicher, 2023), AMPK is an opioid signaling negative feedback loop that is normally activated by opioid at baseline (Veh/DAMGO group) and this activation is disabled by spinal 17-AAG treatment (17-AAG/DAMGO group). NC CRISPR treatment had no impact on this relationship, as expected (Figure 4A). On the other hand, DUSP15 CRISPR treatment blocked this effect, so that pAMPK remained stimulated in the 17-AAG/DAMGO group (Figure 4A); this was confirmed by quantitation (Figure 4B). Taking our previous work on AMPK into account, we would expect this restoration of AMPK stimulation by opioid with DUSP15 knockdown to reinstate the AMPK negative feedback loop and block opioid antinociception. We confirmed this prediction with behavioral experiments. In the tail flick pain model, DUSP15 CRISPR treatment blocked the enhanced morphine pain relief caused by 17-AAG treatment as we showed above; we further treated this group with the AMPK inhibitor dorsomorphin, which restored enhanced opioid antinociception (Figure 4C). We found the same results in the paw incision model (Figure 4D). These findings suggest both that DUSP15 is upstream of AMPK in the Hsp90/MOR signaling cascade, and further that DUSP15 is responsible for the loss of AMPK activation with Hsp90 inhibition. This disables the AMPK negative feedback loop and allows for enhanced morphine signaling and antinociception. Lastly, we noted that both pAMPK (Figure 4A) and DUSP15 (Figure 1B) share a similar staining pattern in cytoplasm/cell bodies in the superficial dorsal horn, unlike the ERK pattern noted above. This could mean that DUSP15 and AMPK are acting in the same region of the same cells.

**FIGURE 4:**
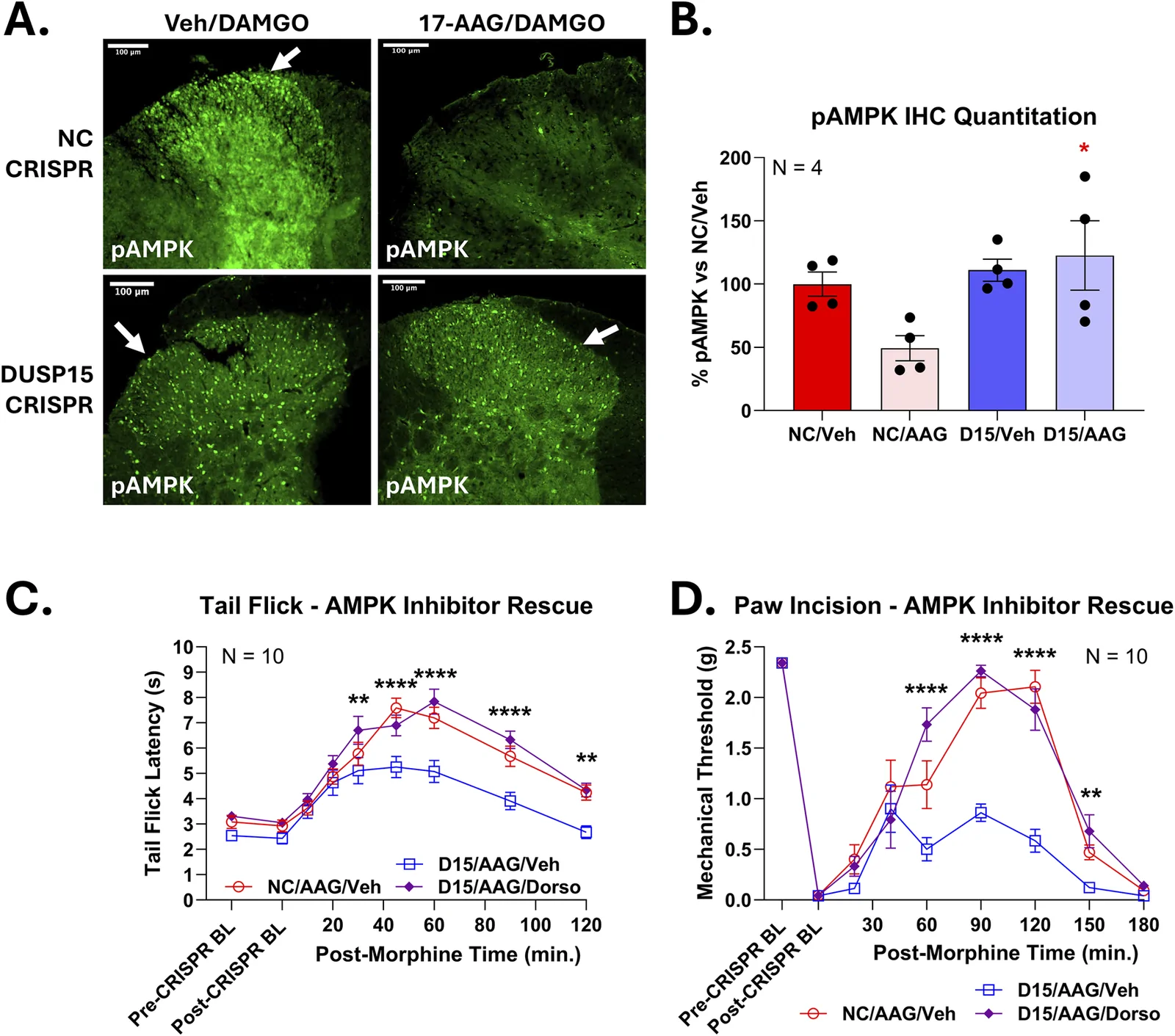
Placement of DUSP15 upstream of AMPK in the Hsp90-MOR signaling cascade. Data represented as the mean ± SEM. **(A)** Male and female CD-1 mice treated with Negative Control (NC) or DUSP15 CRISPR i. t. (see Methods). On day 9, the mice were injected with vehicle or 0.5 nmol 17-AAG i.t., 24 h, then 0.1 nmol DAMGO i.t., 10 min, then sacrificed and their spinal cords analyzed by IHC. N = 4 mice/group with 2 sections per mouse analyzed. Representative images of the dorsal horn region shown. Phosphorylated AMPK (pAMPK) signal (green) was visible as a soma-like stain throughout the dorsal horn region (white arrows). As expected, this signal was lost in the NC CRISPR group with 17-AAG treatment (Gabriel and Streicher, 2023). However, with DUSP15 CRISPR treatment combined with 17-AAG, the pAMPK signal was restored. This observation suggests that DUSP15 is upstream of AMPK, and furthermore acts to deactivate/dephosphorylate the kinase in the Hsp90-MOR cascade (NC/17-AAG group). **(B)** The observations from **(A)** were confirmed by quantitation. The data were further normalized to the NC/Veh group. * = *p* < 0.05 vs. NC/Veh group by 1-Way ANOVA with Tukey’s *post hoc* test. **(C)** Male and female CD-1 mice were treated with NC or DUSP15 CRISPR i.t.; on day 9, the mice were injected with 0.5 nmol 17-AAG i.t., 24 h. On day 10, the mice were injected again with vehicle or 20 nmol of the AMPK inhibitor dorsomorphin i.t., 10 min, then 3.2 mg/kg morphine s. c. and a tail flick time course was performed. Baseline (BL) measurements were also taken. N = 10 mice/group performed in 2 technical replicates. **, **** = *p* < 0.01, 0.0001 vs. same time point D15/AAG/Veh group by RM 2-Way ANOVA with Dunnett’s *post hoc* test. **(D)** A paw incision model was performed using the same experimental design in **(C)**, except that the paw incision surgery was performed on day 9 concurrent with 17-AAG injection, and von Frey filaments were used to measure mechanical thresholds. N = 10 mice/group performed in 2 technical replicates. **, **** = *p* < 0.01, 0.0001 vs same time point D15/AAG/Veh group by RM 2-Way ANOVA with Dunnett’s *post hoc* test. In both pain models, the AMPK inhibitor dorsomorphin reversed the loss of morphine antinociception caused by DUSP15 CRISPR knockdown, confirming the kinase is downstream of DUSP15 and that it’s activated by DUSP15 knockdown to block antinociception.

### DUSP15 regulates opioid antinociception via CGRP neurons

We next sought to identify a cell site of action for DUSP15 signaling. Since we linked DUSP15 to AMPK signaling above, and we found that AMPK signaling in this cascade occurs via CGRP neurons (Gabriel and Streicher, 2023), we hypothesized that DUSP15 is expressed in and regulates opioid antinociception by the same cell type. We tested this hypothesis first using IHC. We stained for DUSP15 and CGRP, finding minimal overlap in the vehicle control group (Figure 5A). However, treatment with 17-AAG caused both an increase in apparent DUSP15 as above, and also a visual increase in DUSP15-CGRP colocalization in the superficial dorsal horn (Figure 5A, yellow, white arrows). This observation was confirmed by a significant increase in the Pearson’s correlation coefficient (Figure 5B). This analysis suggests that upregulated DUSP15 after spinal Hsp90 inhibition does occur at least in part in CGRP neurons.

**FIGURE 5:**
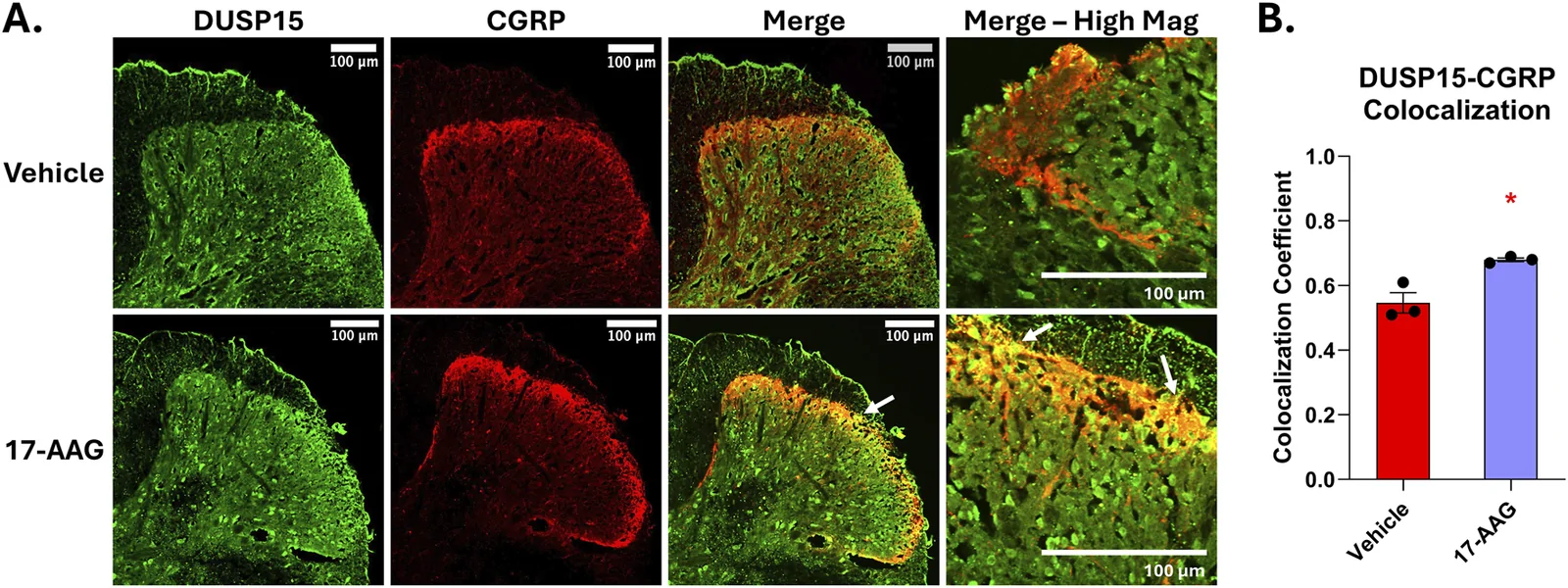
Upregulated DUSP15 colocalizes with CGRP neurons in the superficial dorsal horn of the spinal cord. **(A)** Male and female CD-1 mice were injected with vehicle or 0.5 nmol 17-AAG i.t., 24 h, followed by sacrifice and their spinal cords analyzed by IHC. N = 3 mice/group with 2 slices imaged per mouse. Representative images of the dorsal horn region shown. DUSP15 (green) and CGRP (red) show visible merged overlap (yellow, white arrows) with 17-AAG treatment in the superficial layers of the dorsal horn. This suggests that upregulated DUSP15 is located at least in part in this neuronal population. **(B)** The observations in **(A)** were confirmed by calculating the Pearson’s correlation coefficient between the two channels for each animal. Data shown as the mean ± SEM. * = *p* < 0.05 by unpaired 2-tailed *t*-test. Please note, the Pearson’s coefficient is a scale-independent measure of the strength of correlation, and the measured change cannot be directly converted into a percentage.

However, this colocalization does not demonstrate that DUSP15 regulates opioid antinociception at this site. To do so, we used a modified cell type-selective CRISPR approach that we pioneered (Gabriel and Streicher, 2023); unlike the universal promoter used in the CRISPR experiments above, we used the *Calca* promoter here to selectively drive Cas9 expression in CGRP neurons (i.e., cells in which *Calca* is active). This results in selective gene editing in that cell type ((Gabriel and Streicher, 2023), see Methods). We treated mice with these CRISPR constructs, which we first validated by IHC. NC CRISPR treatment resulted in clear visual DUSP15-CGRP overlap, as above (Figure 6A, white arrows). Treatment with the CGRP-DUSP15 CRISPR, on the other hand, resulted in a loss of DUSP15-CGRP colocalization, with little such signal visible. Importantly, however, DUSP15 signal was not completely erased or gone; clear DUSP15-positive cell bodies were visible throughout the dorsal horn, just not colocalized with CGRP (Figure 6A, red arrow). These visual observations were confirmed by quantitation, which showed a significant loss of colocalization coefficient in the CGRP-DUSP15 CRISPR group (Figure 6B).

**FIGURE 6:**
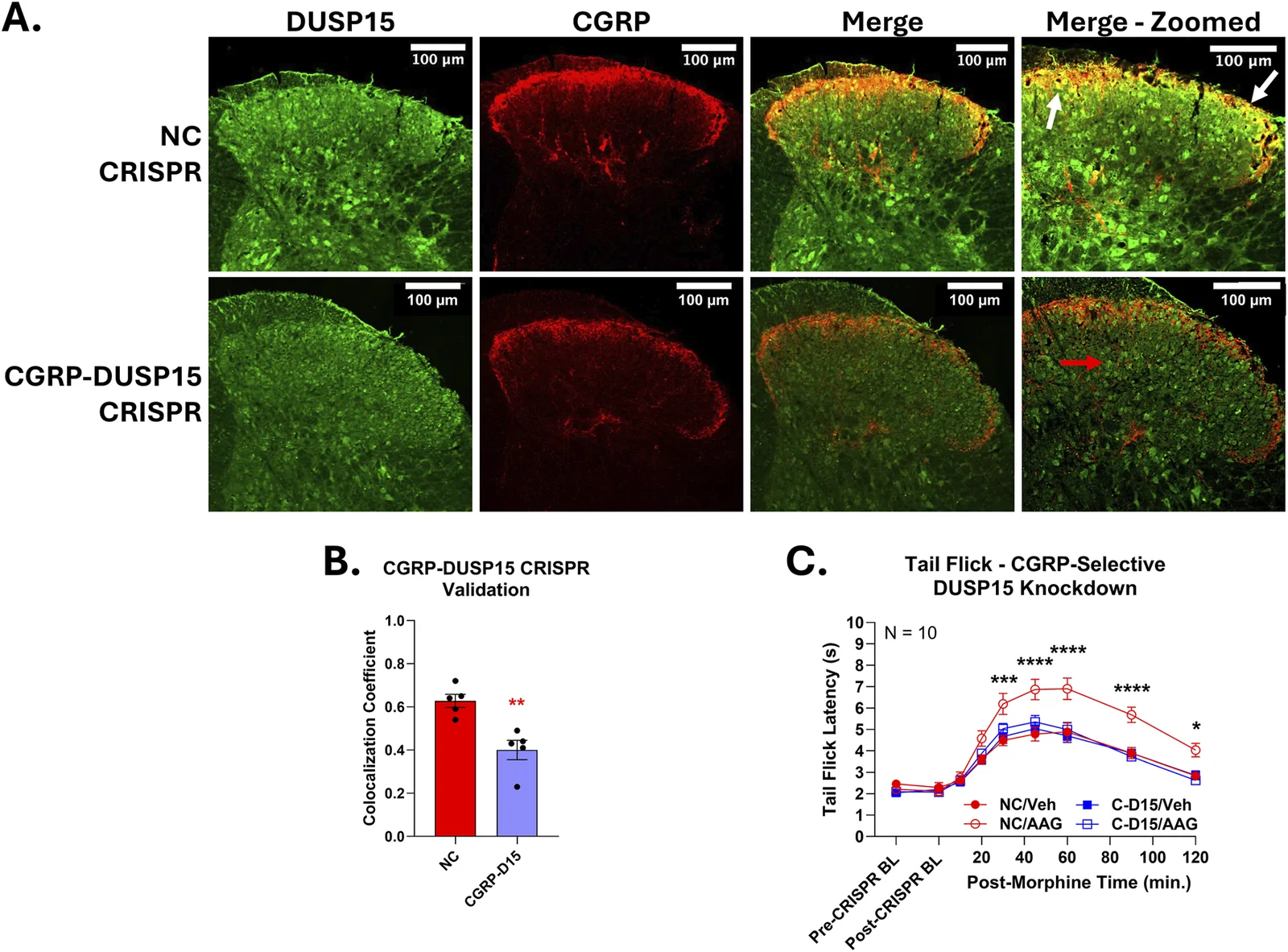
Upregulated DUSP15 within CGRP neurons is necessary for enhanced morphine antinociception after spinal Hsp90 inhibition. Male and female CD-1 mice used for each experiment, and the data was represented as the mean ± SEM. **(A)** The mice were treated with Negative Control (NC) or a CGRP-selective DUSP15-targeted CRISPR knockdown construct (CGRP-DUSP15) by the i.t. route (see Methods). On day 9, all mice were injected with 0.5 nmol 17-AAG by the i.t. route, 24 h, then sacrificed and their spinal cords analyzed by IHC. N = 5 mice/group with 2 sections imaged per mouse. Representative images of the dorsal horn region shown. DUSP15 (green) and CGRP (red) show visible overlap in the superficial dorsal horn (yellow, white arrows) in the NC CRISPR group, as expected. This overlap was visibly lost in the CGRP-DUSP15 CRISPR group. Importantly, however, not all DUSP15 signal was lost; significant non-CGRP-overlapped DUSP15 signal was visible in the tissue (red arrow). This suggested the knockdown was CGRP-selective, as designed. **(B)** The observations from **(A)** were confirmed by comparing the Pearson’s correlation coefficient for both groups. ** = *p* < 0.01 by unpaired 2-tailed *t*-test. **(C)** Thus validated, we used the CGRP-DUSP15 CRISPR in a behavioral experiment. The mice were treated with NC or CGRP-DUSP15 CRISPR as above, and on day 9, injected i.t. with vehicle or 0.5 nmol 17-AAG, 24 h. On day 10, they were injected with 3.2 mg/kg morphine s. c. and a tail flick time course was performed; baseline (BL) measurements were also taken. N = 10 mice/group performed in 2 technical replicates. *, ***, **** = *p* < 0.05, 0.001, 0.0001 vs. same time point NC/Veh group by RM 2-Way ANOVA with Dunnett’s *post hoc* test. The CGRP-DUSP15 CRISPR treatment reversed the enhancement caused by 17-AAG, much like the non-selective CRISPR treatments above, demonstrating the necessity of DUSP15 in CGRP neurons for the Hsp90-MOR signal transduction cascade.

Thus validated, we proceeded to use our CGRP-DUSP15 CRISPR in a behavioral experiment. We combined spinal CRISPR with 17-AAG and morphine treatments in the same experimental design as Figure 1. We observed that the CGRP-DUSP15 CRISPR treatment reversed the enhanced morphine antinociception caused by 17-AAG perfectly back to baseline (NC/Veh) (Figure 6C). This experiment showed the same pattern as the universal CRISPR experiment in Figures 1D,E. This observation ties together our findings and suggests that DUSP15 upregulation with spinal Hsp90 inhibition occurs in CGRP neurons, and through this site of action the protein promotes enhanced opioid pain relief.

## Discussion

Our findings here build on a model of Hsp90-regulated MOR signal transduction in the spinal cord. We previously found that spinal Hsp90 inhibition enhances ERK activation by the MOR (Duron et al., 2020) and disables an AMPK negative feedback loop in CGRP neurons (Gabriel and Streicher, 2023), both of which lead to enhanced opioid antinociception, which itself could be the basis of a means to improve opioid therapy (Duron et al., 2024). Notably, the first two studies and this study used the non-isoform-selective inhibitor 17-AAG while the last report dissected out which isoforms regulate opioid antinociception; this means that most of our analysis and this report do not distinguish between Hsp90 isoforms. In this report we extend this model further by finding that DUSP15 is upregulated in least in part in CGRP neurons, and that through this site of action DUSP15 enhances opioid pain relief. Furthermore, DUSP15 is upstream of both ERK and AMPK in the Hsp90/MOR signaling cascade, and shares a site of action in CGRP neurons with AMPK. Together, our findings here combined with our previous work suggest a model whereby DUSP15 disables AMPK signaling, which removes a negative feedback brake on ERK signaling, leading to enhanced opioid antinociception. This model is diagrammed in Figure 7. This report is also the first to describe a role for DUSP15, or any DUSP, in opioid antinociceptive signaling.

**FIGURE 7:**
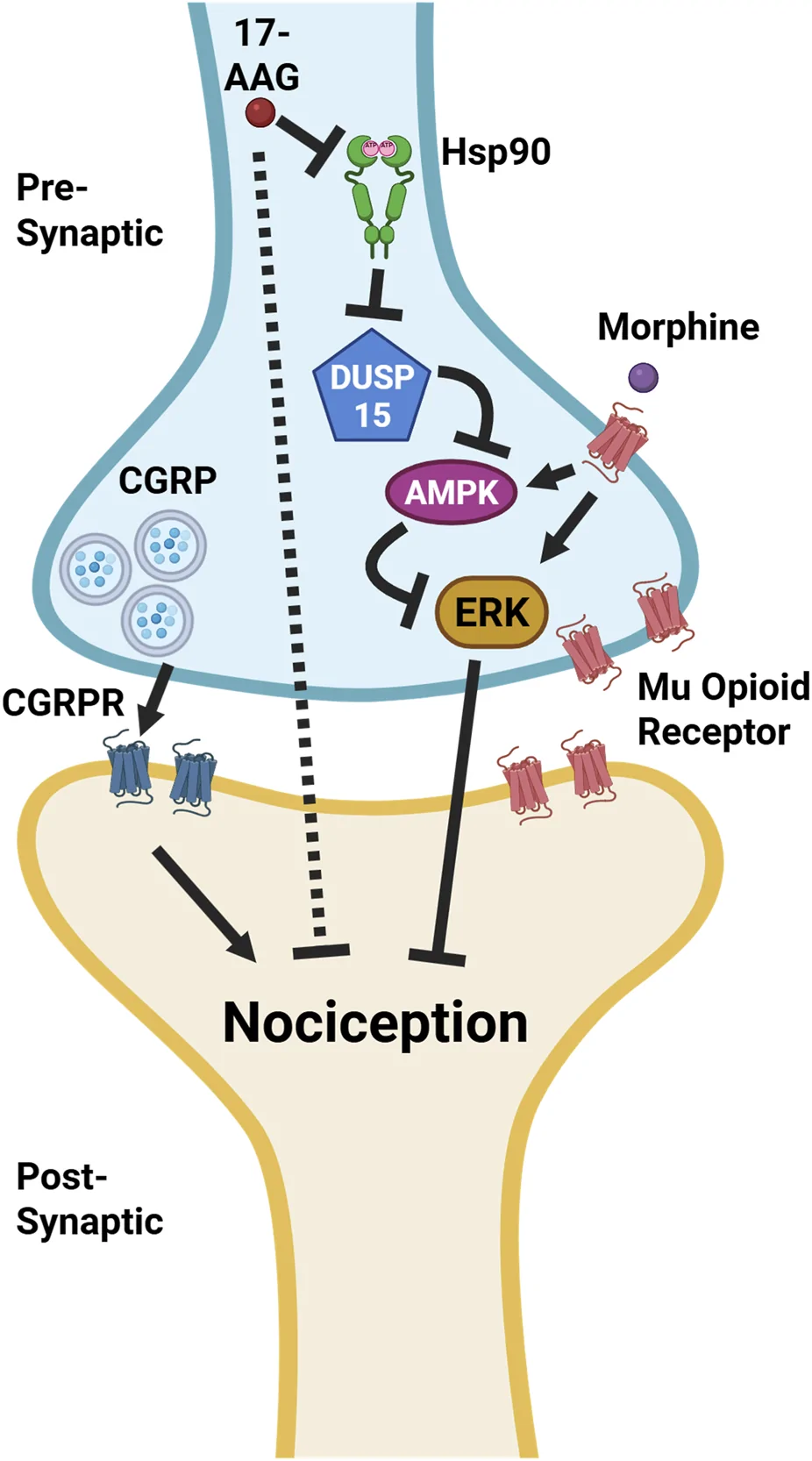
Summary model of the Hsp90-MOR signal transduction cascade. Inhibition of Hsp90 with 17-AAG relieves some sort of blockade or prevention of DUSP15 expression, leading to enhanced DUSP15 levels in CGRP neurons. This leads to dephosphorylation of AMPK, which itself relieves a negative feedback brake on ERK, leading to enhanced ERK signaling and enhanced morphine antinociception. DUSP15 and AMPK signaling within CGRP neurons is necessary for this cascade to take place. Overall, this enhancement in opioid antinociception could be used to improve opioid therapy. Figure created using biorender.com.

The mechanism by which DUSP15, a phosphatase, promotes kinase phosphorylation and signal transduction is not clear. Earlier studies showed that DUSP15 promotes ERK MAPK and STAT3 phosphorylation, although the mechanism was not revealed in these studies (Rodriguez-Molina et al., 2017; Kikkawa et al., 2024). In contrast, overexpression of DUSP15 in the nucleus accumbens abolished ERK MAPK activation in response to morphine conditioning, demonstrating both that DUSP15 can promote *de-*phosphorylation, and also that it can oppositely regulate the same signaling kinase (ERK) (Qiao et al., 2021). However, our work does suggest how this might happen in the spinal cord opioid antinociception context. Our earlier analysis placed AMPK upstream of ERK in the Hsp90-MOR cascade (Gabriel and Streicher, 2023). Our findings here place DUSP15 upstream of both (Figures 3,4). Combining these analyses suggests a DUSP15-AMPK-ERK signaling pathway; further, our earlier study strongly suggested that AMPK acts as a negative feedback brake on ERK, preventing ERK phosphorylation (Gabriel and Streicher, 2023). Since DUSP15 appears to prevent AMPK phosphorylation (Figure 4), the most parsimonious model is that after Hsp90 inhibition, DUSP15 directly dephosphorylates AMPK, which relieves the negative brake on ERK, leading to ERK phosphorylation and enhanced opioid antinociception (Figure 7). DUSP15 has not been studied in the context of AMPK activity, however, DUSP1 was found to dephosphorylate AMPK, lending feasibility to our model (Sanders et al., 2022). Care should be taken to not over-interpret this precedent, however, since both DUSP14 and DUSP29 were found to promote AMPK phosphorylation (Cooper et al., 2020; Zhao et al., 2021). Clearly the roles of this phosphatase family can be quite heterogenous, and likely depend on local regulatory factors and organization in different cellular and signaling contexts to lead to a particular outcome (Streicher, 2019).

Our combined findings also begin to suggest a cell/circuit mechanism by which this signaling cascade occurs. The reasoning laid out above suggests that DUSP15 and AMPK could directly interact. Our immunohistochemistry observations support this hypothesis. Activated AMPK signals via a soma pattern in CGRP-expressing neurons in the dorsal horn (Figure 4 (Gabriel and Streicher, 2023)); our experiments here show a similar soma/CGRP pattern for DUSP15 (Figures 5,6). This places both molecules in proximity for the proposed dephosphorylation of AMPK by DUSP15, and our experiments further show that this CGRP location is necessary for enhanced opioid antinociception by both molecules. Importantly, however, our data does not show direct dephosphorylation of AMPK by DUSP15, which must still be demonstrated, and an intermediate factor could be present. Since the molecules are found in CGRP somata, this strongly suggests that this part of the cascade must occur in the 2° nociceptors or below (CGRP 1° nociceptor cell bodies are in the dorsal root ganglion); furthermore, these CGRP interneurons have been shown to contribute to mechanical hypersensitivity (Loken et al., 2021). Notably, our studies did not define possible CGRP sub-populations, which should be examined in future studies. Our combined experiments show that ERK is downstream of both DUSP15 and AMPK per above, and our earlier study showed that this phosphorylated ERK colocalized with the dendrite marker MAP2 in the superficial dorsal horn (Duron et al., 2020). This would suggest that the ERK signaling event takes place downstream of the CGRP neurons signaling with DUSP15 and AMPK, in the 3° nociceptors or below. This separates the DUSP15/AMPK signaling from the ERK signaling both in space and potentially across different cells. What links AMPK and ERK across this physical gap in the Hsp90-MOR cascade is not known, and should be uncovered in future studies. It’s also not clear how enhanced ERK signaling contributes to enhanced opioid antinociception, with no analogous spinal cord studies to be found in the literature; given the signaling locations in CGRP nociceptors, this may occur via suppressing CGRP nociceptor neuronal activity and/or CGRP release, or could occur through a different mechanism. Certainly, MOR signaling generally has been shown to accomplish both (Ballet et al., 1998). These details must also be uncovered by future work, and this caveat must be kept in mind since such studies were not performed here, along with other possible details like subtle sex-dependent differences and the role of specific Hsp90 isoforms.

It’s also not yet clear what mechanism links Hsp90 activity to DUSP15 expression in CGRP neurons. Tonic high levels of DUSP15 expression were observed in the very deep layers of the dorsal horn, close to the intermediate horn region, and this expression was not altered by Hsp90 inhibition (Figure 1B). Hsp90 regulation of DUSP15 expression may thus vary by cell context, with the CGRP neurons of the superficial dorsal horn showing this relationship while other contexts do not. No studies have linked Hsp90 to DUSP15 or any other DUSP to our knowledge; the closest appears to be one report which showed that the extracellular isoform of Hsp70 indirectly enhanced DUSP1 expression via the glucocorticoid receptor (Zhou et al., 2023). Certainly, Hsp90 can directly stabilize the expression of multiple client proteins, and Hsp90 inhibition can release various client transcription factors, such as Heat Shock Factor-1, which could lead to increased transcription and expression of DUSP15 (Li and Buchner, 2013). Hsp90 could also change DUSP15 expression by an indirect mechanism, such as altering the signaling of other receptor systems which in turn regulate DUSP15 expression (Streicher, 2019). Our previous proteomic study, in addition to identifying the upregulation of DUSP15 (Figure 1A), also identified altered proteins in processes such as transcription (YAP1) and signaling (Phospholipase C), providing potential signposts to mechanisms for future investigation (Duron et al., 2020). Specific Hsp90 isoforms could also have specific roles, which was elided by the use of a non-selective inhibitor in this study.

These findings may also lead to methods to improve opioid therapy. Our previous work showed that Hsp90 had opposite effects on opioid signaling in brain vs. spinal cord, so that Hsp90 inhibition in brain blocked opioid pain relief while inhibition in the spinal cord enhanced it (Lei et al., 2017; Duron et al., 2020). We linked these signaling outcomes to different Hsp90 isoforms, with Hsp90α alone regulating opioid signaling in the brain (Lei et al., 2019), while Hsp90α, Hsp90β, and Grp94 all regulated opioid signaling in the spinal cord (Duron et al., 2024). This led to our discovery that Hsp90β- or Grp94-selective inhibitors could be given by a translationally relevant route of administration (e.g., orally) to improve the therapeutic index of opioids, both enhancing pain relief and decreasing side effects (Duron et al., 2024). The Hsp90 inhibitors were effective in acute (tail flick), acute/pathological (paw incision), and chronic (HIV-induced peripheral neuropathy) pain models, suggesting a broad shared mechanism for Hsp90 and DUSP15 across multiple pain types. Granted, DUSP15 was not tested in the chronic pain model, so in principle, it may differ, and should be tested in the future, along with which specific isoforms regulate DUSP15. However, the mechanisms uncovered here may also be impactful in improving opioid therapy. No DUSP15 ligands exist to our knowledge, however, the compound BCI is a DUSP1/6 inhibitor, providing proof-of-principle that the family can be pharmacologically targeted (Korotchenko et al., 2014). Our observations suggest that a DUSP15 activator/agonist could provide much the same benefit as an isoform-selective Hsp90 inhibitor, with perhaps a lower risk profile due to the more constrained expression and role of DUSP15 vs. Hsp90, although this was not directly tested by our study. In addition, these studies provide an improved basic science foundation to our effort to target Hsp90 and other MOR signaling molecules in the spinal cord to improve opioid therapy.

## Data Availability

The original contributions presented in the study are included in the article/Supplementary Material, further inquiries can be directed to the corresponding author.
