# Supplementary material for: Inhibition of heat shock protein 90 in the mouse spinal cord enhances opioid antinociception via the upregulation of dual specificity protein phosphatase 15 (DUSP15) in CGRP neurons
Source: Front Pharmacol. 2026 Oct 19;17:1734504. doi: 10.3389/fphar.2026.1734504 (PMC13724451; doi:10.3389/fphar.2026.1734504)
Supplement: Supplementary file 1 [file DataSheet1.pdf]

Supplementary Information

for

Inhibition of Heat shock protein 90 in the Mouse Spinal Cord Enhances Opioid Antinociception via the Upregulation of Dual Specificity Protein Phosphatase 15 (DUSP15) in CGRP Neurons

Katherin A. Gabriel[1], Jessica L. Bowden[1], and John M. Streicher[1,2,*]

[1]Department of Pharmacology, College of Medicine, University of Arizona, Tucson AZ USA

[2]Comprehensive Center for Pain and Addiction, University of Arizona, Tucson AZ USA

***Corresponding Author:** John M. Streicher, Ph.D.; University of Arizona College of Medicine, Box 245050, LSN563, 1501 N. Campbell Ave., Tucson AZ 85724. Ph: (520)-626-7495. Email: jstreicher@arizona.edu

ORCID: John Streicher 0000-0002-4173-7362

Keywords: Heat shock protein 90 (Hsp90); opioid; antinociception; Dual specificity protein phosphatase 15 (DUSP15); calcitonin gene-related peptide (CGRP)

Running Title: Hsp90-DUSP15 Signaling in Spinal Cord

Number of Figures: 7 (plus 2 Supplemental Figures)

Figure S1

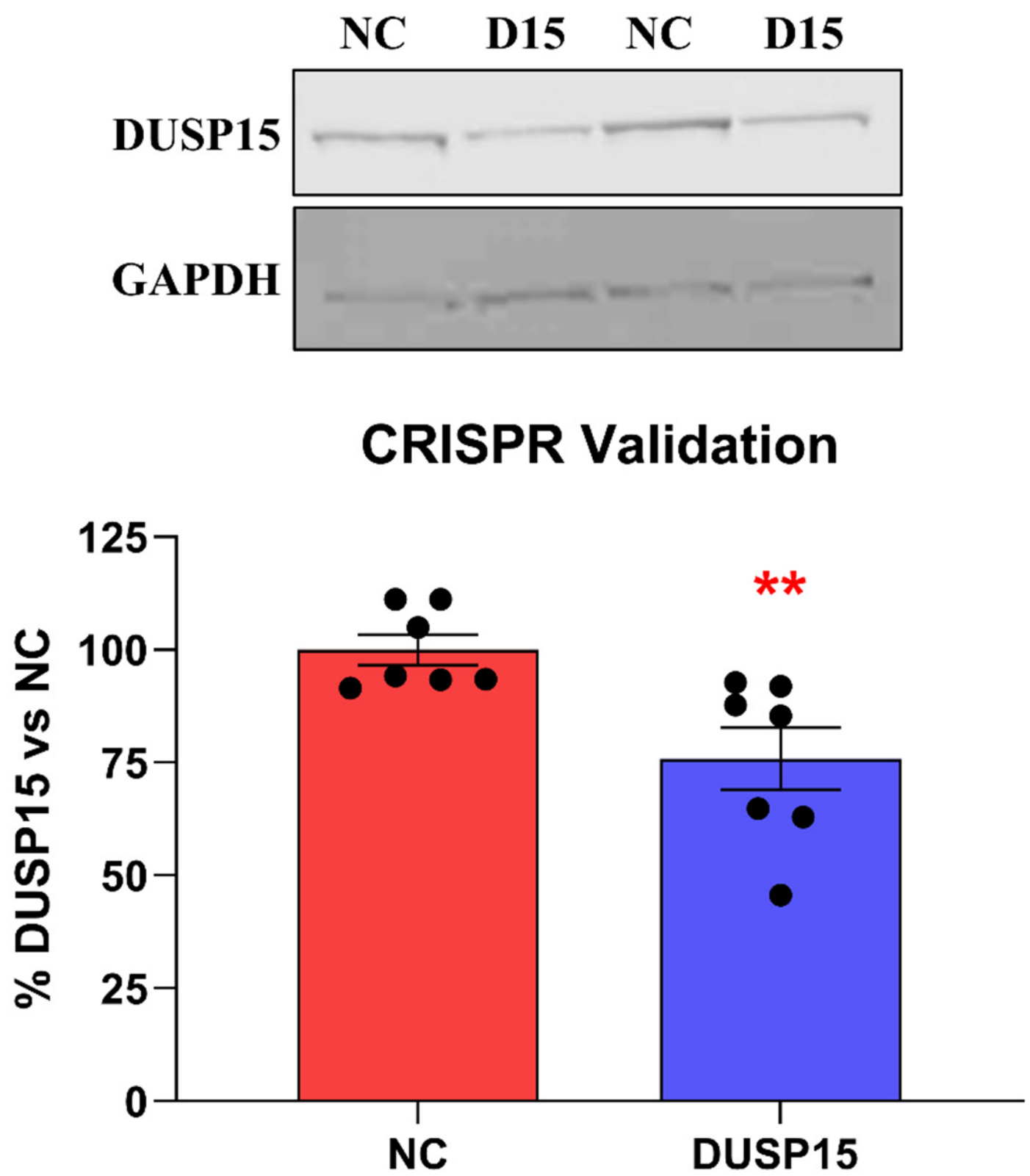

Figure S1: Validation of non-selective DUSP15 CRISPR knockdown in mouse spinal cord. Male and female CD-1 mice were treated with Negative Control (NC) or DUSP15-targeted CRISPR by the i.t. route (see Methods). On day 10, the spinal cords were removed and analyzed by Western blot for DUSP15 and GAPDH loading control protein levels. N = 7 mice/group, with the data shown as the mean ± SEM. *Top* – representative blots shown for DUSP15 and GAPDH. *Bottom* – quantitation of the full data set. Each DUSP15 band was normalized to the GAPDH control from the same sample on the same blot, and the data were further normalized to the NC group. ** = $p < 0.01$ by unpaired 2-tailed t test. This experiment validated the effectiveness of the CRISPR knockdown at the protein level.

Figure S2

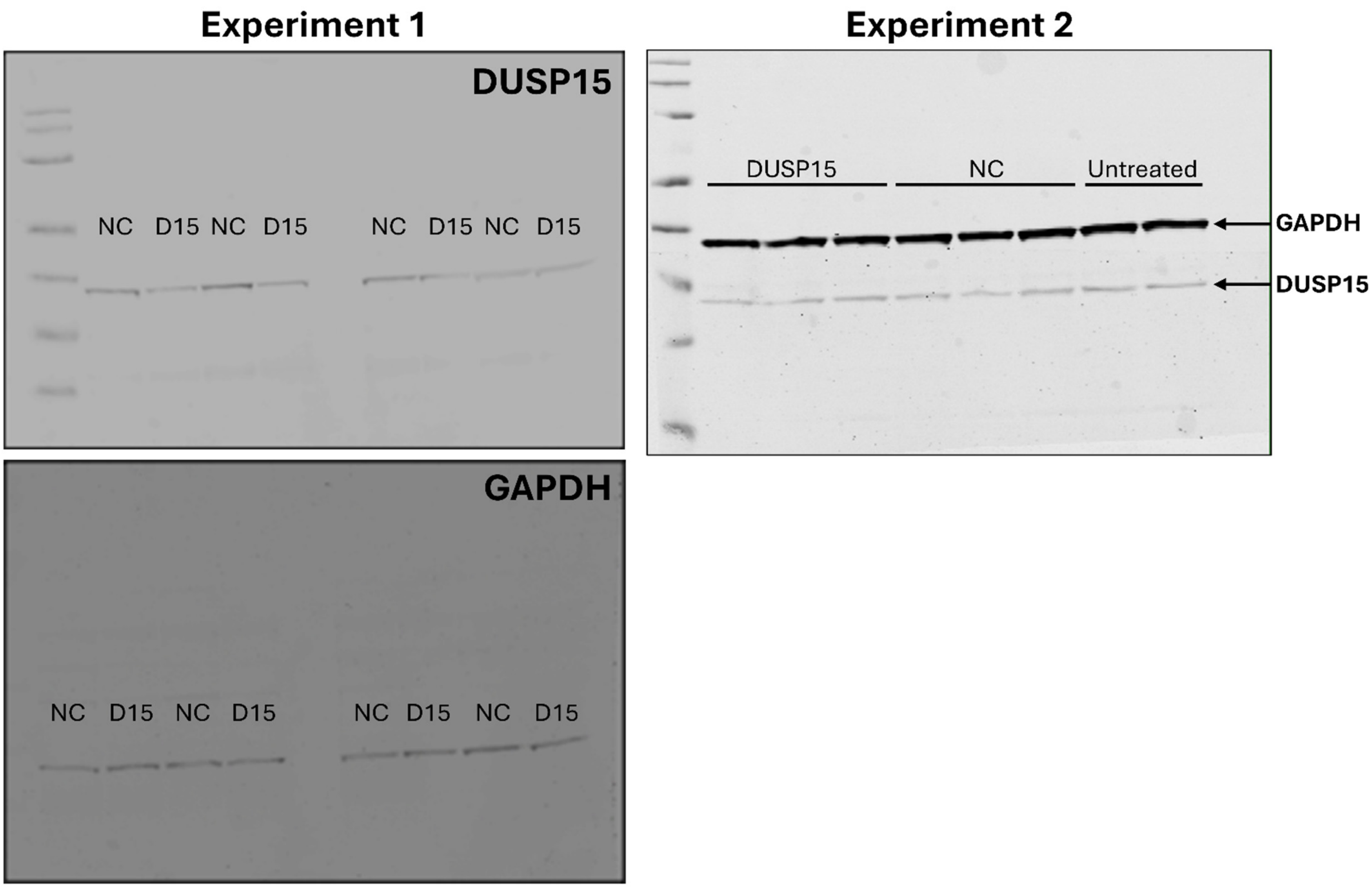

Figure S2: Original Western blot images for Figure S1.
